# Air Kerma Calculation in Diagnostic Medical Imaging Devices Using Group Method of Data Handling Network

**DOI:** 10.3390/diagnostics13081418

**Published:** 2023-04-14

**Authors:** Licheng Zhang, Fengzhe Xu, Lubing Wang, Yunkui Chen, Ehsan Nazemi, Guohua Zhang, Xicai Zhang

**Affiliations:** 1 College of Information Engineering, Zhejiang University of Technology, Hangzhou 310014, China; 2 Department of Digital Media Technology, Hangzhou Dianzi University, Hangzhou 310018, China; 3 Department of Radiology, Taizhou First People’s Hospital, Taizhou 318000, China; 4 Department of Physics, University of Antwerp, 2610 Antwerp, Belgium; 5 Department of General Surgery, Pingyang Hospital Affiliated to Wenzhou Medical University, Wenzhou 325000, China

**Keywords:** GMDH neural network, X-ray tube, medical diagnostic radiology, air kerma

## Abstract

The air kerma, which is the amount of energy given off by a radioactive substance, is essential for medical specialists who use radiation to diagnose cancer problems. The amount of energy that a photon has when it hits something can be described as the air kerma (the amount of energy that was deposited in the air when the photon passed through it). Radiation beam intensity is represented by this value. Hospital X-ray equipment has to account for the heel effect, which means that the borders of the picture obtain a lesser radiation dosage than the center, and that air kerma is not symmetrical. The voltage of the X-ray machine can also affect the uniformity of the radiation. This work presents a model-based approach to predict air kerma at various locations inside the radiation field of medical imaging instruments, making use of just a small number of measurements. Group Method of Data Handling (GMDH) neural networks are suggested for this purpose. Firstly, a medical X-ray tube was modeled using Monte Carlo N Particle (MCNP) code simulation algorithm. X-ray tubes and detectors make up medical X-ray CT imaging systems. An X-ray tube’s electron filament, thin wire, and metal target produce a picture of the electrons’ target. A small rectangular electron source modeled electron filaments. An electron source target was a thin, 19,290 kg/m^3^ tungsten cube in a tubular hoover chamber. The electron source–object axis of the simulation object is 20° from the vertical. For most medical X-ray imaging applications, the kerma of the air was calculated at a variety of discrete locations within the conical X-ray beam, providing an accurate data set for network training. Various locations were taken into account in the aforementioned voltages inside the radiation field as the input of the GMDH network. For diagnostic radiology applications, the trained GMDH model could determine the air kerma at any location in the X-ray field of view and for a wide range of X-ray tube voltages with a Mean Relative Error (MRE) of less than 0.25%. This study yielded the following results: (1) The heel effect is included when calculating air kerma. (2) Computing the air kerma using an artificial neural network trained with minimal data. (3) An artificial neural network quickly and reliably calculated air kerma. (4) Figuring out the air kerma for the operating voltage of medical tubes. The high accuracy of the trained neural network in determining air kerma guarantees the usability of the presented method in operational conditions.

## 1. Introduction

There are two steps in the process by which photons impart their energy to matter. The interaction of photons with matter first transfers energy to the charge carriers of matter. The charge carriers’ kinetic energy is then deposited by the ionized and excited atoms. By dividing the total kinetic energy of the charged particles (such as electrons, protons, and other charged atoms) that are released when the rays impact something, we may obtain a measure of the radiation that goes through that item; this measure is called the kerma. Kinetic energy divided by matter mass yields this value [1]. Ionizing radiation without a charge is referred to as “kerma” by scientists. The quantity of radiation that has been absorbed is equal to the amount of kerma, which is measured in gray. A mass of air has the same amount of kerma as another mass of air. As measuring air kerma is much easier than measuring the dosage, it is often used for radiation equipment calibration [2]. In interventional radiology, if the skin dosage is high enough to induce radiographic burns to the patient, air kerma computation is also used to forecast the skin dose [3]. Researchers have recently been interested in studying the air kerma created by X-ray tubes. Another article looked at how changing the anode angle or the wave voltage of the X-ray tube affected the air kerma. A Philips MCN165 was used to test the X-ray tube model at a voltage range of 40 to 140 kV [4]. In this investigation, it was found that raising the anode angle had the same effect on airflow as raising the supply voltage. They also claim that the air kerma lessens the severity of wrinkles. After introducing the Monte Carlo simulator for a sodium iodide detector, Oliveira et al. [5] developed a spectral separation method for determining the air kerma from X-rays. Without the suggested spectrum stripping procedure, the discrepancy between the derived spectrum and the reference spectrum was over 63%, but it was reduced to less than 0.2%. The kerma of the chest wall in kids and teens was investigated by Porto et al. [6]. According to the findings of this study, air tension falls as tube voltage rises and exposure falls. Air kerma has been measured and reported on by researchers in the medical and industrial sectors [7,8,9,10,11,12,13,14,15]. These analyses did not include the rest of the X-ray tube’s radiation field in their estimation of air kerma at the tube’s core. It should be noted that the quantity of air kerma changes with the angle inside the X-ray beam, even when the anode is kept at a constant distance. The anode heel effect is the source of this discrepancy. These analyses did not include the rest of the X-ray tube’s radiation field in their estimation of air kerma at the tube’s core. Notably, the quantity of air kerma changes with distance from the anode in the radiation field. The anode heel effect is to blame for the discrepancy between these values. Some researchers have investigated the heel effect in the radiation field. In Ref. [16] researchers have tried to determine air kerma using an intelligent method. Although they used the MLP neural network to predict air kerma, the accuracy of the methodology they presented in air kerma prediction was not high. In the next research [17], the researchers investigated the performance of the RBF neural network for forecasting air kerma. Although the accuracy increased, it is predicted that by selecting the appropriate neural network, the accuracy in determining air kerma can be increased even more. Despite the existence of the anode heel effect, this research presents a technique for accurately estimating air kerma. The air kerma was computed and simulated using the Monte Carlo N Particle (MCNP) algorithm at six different X-ray tube voltages and various distances to the source. Using the MCNP code’s sparse data, a Group Method of Data Handling (GMDH) neural network is trained to generate predictions about the air kerma. The trained neural network can calculate the air kerma for any given X-ray tube voltage and position in the X-ray field. While the MCNP algorithm may be used to calculate air kerma, this is a time-consuming procedure, hence it is more efficient to employ a neural network to make predictions about air kerma. The present investigation is organized as follows: A thorough description of the structure that the MCNP algorithm simulates is provided in Section 2. In the next section, these simulation data are used to teach the GMDH neural network. The findings and conclusions are presented in Section 4 and Section 5, respectively.

The following are some of the major findings of this study.

1. The heel effect is taken into account while calculating air kerma.
2. Calculating the air kerma by employing an artificial neural network and training it with a limited amount of data in varying angles, distances, and voltages of tubes.
3. Using an artificial neural network, the calculation of air kerma was executed extremely quickly and accurately compared to earlier efforts.
4. Calculating the air kerma for medical tubes’ operating voltage.

## 2. Methodology

**Figure 1:**
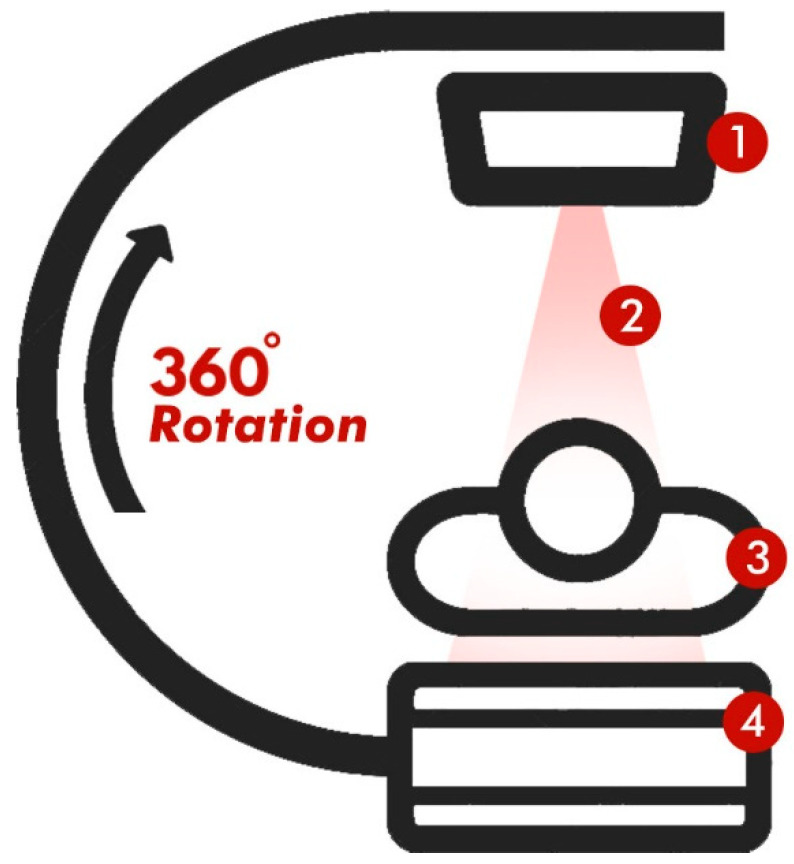
A medical X-ray computed tomography imaging system: (1) X-ray tube, (2) conical X-ray beam, (3) patient, and (4) detector.

**Figure 2:**
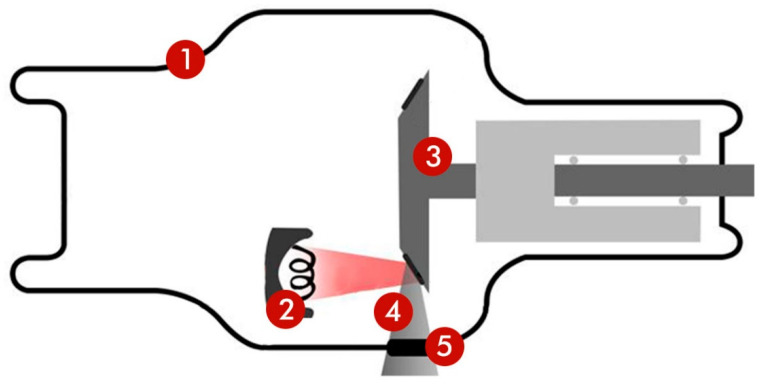
The X-ray tube: (1) shield, (2) electron filament, (3) target, (4) X-ray radiation beam, and (5) window.

As shown in Figure 1, the two main components of a medical X-ray CT imaging system are the X-ray tube and the detector. An X-ray tube’s electron filament (a thin wire) and metal target allow for the production of an X-ray image (the object the electrons hit). After being generated by the filament, electrons are propelled through a large potential difference in the X-ray source’s hoover chamber before striking the target. The Bremsstrahlung process converts just a small proportion of the energy in electrons into photons, therefore most of the energy ends up as heat. Several projections, or 2D pictures, are taken when the X-ray tube and detector spin around the subject at the same time. The system takes 2D photos of the patient and uses powerful computer technology to recreate 3D images of their body in accordance with Lambert Beer’s law. In the medical X-ray imaging sector, air kerma has only been studied using a model of an X-ray tube (as shown in Figure 2). A medical X-ray tube is simulated using code written in MCNPX. To model electron filaments, a tiny rectangular electron source was examined. If you want to simulate focal spots, you will need to use a surface source rather than a point source. A thin tungsten cube with a density of 19,290 kg/m^3^ was placed in a tubular vacuum chamber as an electron source target. The electron source–object axis of the simulation object has an angle of 20° to the vertical. It is important to note that at the maximum tilt angle of the X-ray tube target, the emitted X-rays leave the tube within the cone. To create the illusion of a hoover chamber, the electrons and the target are encased in a steel shell. The only section of the hoover chamber that has any action was the exposed circle. At the entrance to the vacuum chamber is a beryllium window with a density of 1850 kg/m^3^ and a thickness of 1 mm. Two-stage point detector counting was used to determine air kerma (tally F5). At each detector, the photon flux was first measured. The floating air kerma conversion factor recommended in the ICRP-51 report of the International Committee on Radiation Protection was used to determine the air kerma in the second stride [18]. It should be noted that the overall statistical uncertainty did not exceed 4% in all Monte Carlo simulations in this study. In this article, we employ a spherical coordinate system to precisely locate point detectors (according to the inherent spherical symmetry of the X-rays produced) at various tangent angles (0°, 2°, 4°, 6°, 8°, and 10°, point detectors were placed at 25, 50, 75, 100, and 125 mm from the source at 12°, 14°, 16°, 18°, and 20°, respectively) and polar angles (Φ = 0°, 15°, 30°, 45°, 60°, 75°, 90°, 105°, 120°, 135°, 150°, 165°, 180°, 195°, 210°, 225°, 240°, 255°, 270°, 285°, 350°, 330°, 345°, and 360°). The air kerma was calculated from the installed control points for tube voltages of 40, 60, 80, 100, 120, and 140 kV. Calculating the air kerma map for a given set of parameters required about 96 h when using a personal computer with an Intel(R) Core(TM) i7 CPU and 8GB RAM for Monte Carlo simulation.

### GMDH Neural Network

In recent years, researchers have used mathematical models called artificial neural networks to help them understand how radiation interacts with tissue [18,19,20,21,22,23,24,25,26,27,28,29,30,31]. Moreover, the strong mathematical tool of numerical computing [32,33,34,35,36,37,38] has been employed to solve various engineering challenges, most notably in the field of artificial networks [39,40,41,42,43,44]. One of the intelligent methods for solving complex and nonlinear problems was developed in 1968 by M.G. Ivakhnenko and named GMDH [45]. In fact, these algorithms create self-examination methods with prediction, classification, control synthesis, and system debugging capabilities. The characteristics of the network structure, including the number of layers, the number of important input features, and the ideal network configuration, were all detected automatically using Ivahnenko’s method. This method presumes that Kolmogorov–Gabor polynomials of higher order determine the system’s input and output equations.

(1) $$y=a_{0}+\sum_{i=1}^{m}a_{i}x_{i}+\sum_{i=1}^{m}\sum_{j=1}^{m}a_{ij}x_{i}x_{j}+\sum_{i=1}^{m}\sum_{j=1}^{m}\sum_{k=1}^{m}a_{ijk}x_{i}x_{j}x_{k}+\cdots$$

x(x_1_, x_2_, …, x_m_) represents the input (the features vector), a(a_1_, a_2_, …, a_m_) represents the coefficient or weight, and y(x) represents the network’s output. The following procedures should have been carried out in order to use a GMDH network:

In the first step, new variables should have been created and quadratic regression polynomials are calculated based on Equation (2) for each combination and two at a time for all characteristics (x_1_, x_2_…x_m_).

(2) $$Z=c_{1}+c_{2}x_{i}+c_{3}x_{j}+c_{4}x_{i}^{2}+c_{5}x_{j}^{2}+c_{6}x_{i}x_{j}$$

Coefficient C was determined using the least squares method in this investigation. Take note of how each of the quadratic polynomials computed is quite close to the target value. A quadratic polynomial is calculated by each neuron. Secondly, dead neurons are those that could not accurately forecast the required product. The leftover neurons are employed for the layer-up procedure. This process not only creates the first neural layer, but also chooses the most effective neurons. The third phase involves using the polynomial found in the second stage to generate the next layer. This means that the old polynomial is used as a basis for creating a new polynomial, and the second step is repeated until an effective neuron is located. The GMDH neural network is not complete until this process is repeated several times. In the final stage, accuracy is guaranteed and test data are used to assess the efficiency of the designed network. Training data and test data are created throughout the neural network construction phases. The training data are used to create the neural network, and the error is minimized by tuning the network’s various parameters. After the training process is complete, the network’s effectiveness ought to be evaluated against data it has never seen before to ensure it has retained what it has learned. If this step is completed successfully, the network will behave as expected under operating circumstances. Around 70% of the data were used for training and the remaining 30% of the data were used for evaluation. 

## 3. Results

**Figure 3:**
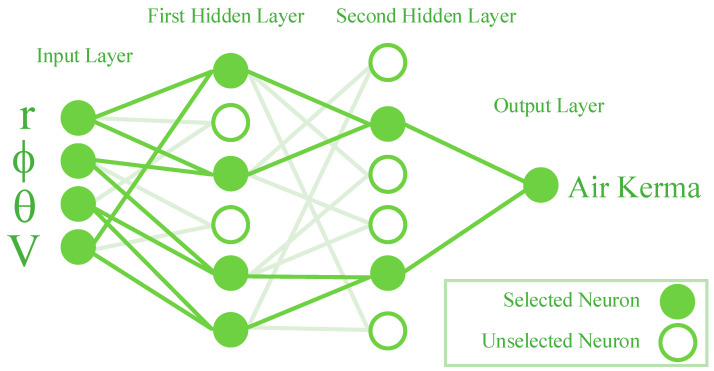
The architecture of the proposed GMDH neural network.

For this research, the air kerma was calculated using a GMDH neural network. After extracting the function, it was fed into the network. For accurate air kerma estimation, the functions ϕ, θ, r, and V were found to be most useful. Figure 3 depicts the intended structure of the GMDH neural network.

The GMDH network took into consideration the voltage of the X-ray tube and the position inside the radiation field as inputs. Output was measured in kerma of air. A neural network was trained by randomly picking 5775 samples from the provided data. When training was complete, the remaining data was utilized to evaluate the neural network. Two hidden layers, each with 4 neurons, were able to give accurate correlations between inputs and outputs. Two error measures, mean relative error (MRE) and root mean square error (RMSE), were used to determine the discrepancy between the MCNP code’s air kerma volume and the neural network’s air kerma prediction. These requirements are represented by the following equations:

(3) $$MRE\%=100\times \frac{1}{N}\sum_{j=1}^{N}\left|\frac{X_{j}\left(Exp\right)-X_{j}\left(Pred\right)}{X_{j}\left(Pred\right)}\right|$$

(4) $$RMSE={\left[\frac{\sum_{j=1}^{N}{\left(X_{j}\left(Exp\right)-X_{j}\left(Pred\right)\right)}^{2}}{N}\right]}^{0.5}$$

X(Exp) and X(Pred) are the experimental and predicted values, whereas N is the total number of samples. 

## 4. Discussion

**Figure 4:**
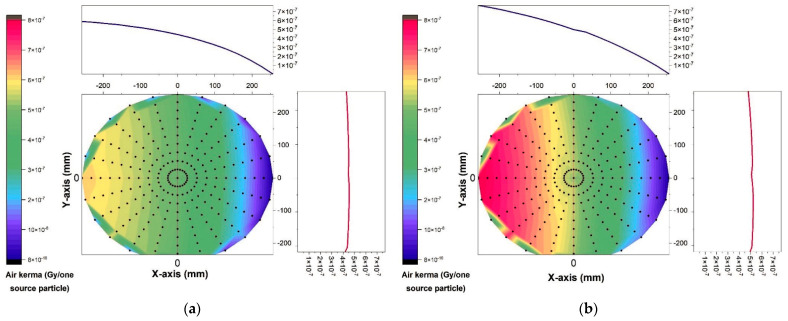
The air kerma determined with the help of the Monte Carlo method at (**a**) 60 kV and (**b**) 120 kV.

**Figure 5:**
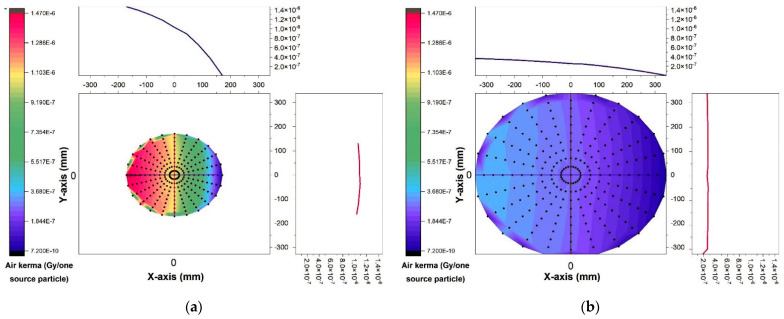
The air kerma determined with the help of the Monte Carlo method at (**a**) 500 mm and (**b**) 1000 mm.

The air kerma, calculated using the Monte Carlo model, is shown in Figure 4 for two different tube voltages (60 kV and 120 kV) based on the X-ray field of view at a distance of 75 mm from the source. Figure 4a,b demonstrate how the heel effect of the X-ray tube causes the anticipated air kerma to be smaller on the right side of the field of view (towards the target) than on the left, despite being almost uniform from top to bottom. The kerma of the air increases as the voltage in the tube increases. To demonstrate the effect of distance, the air kerma is computed with the voltage held constant at 80 kV and the detector placed at distances of 500 mm and 1000 mm, respectively, in Figure 5a,b. Air kerma drops down dramatically with distance from the source, as predicted. This implies if the radiation source travels away from the treated region, the quantity of radiation that reaches that area goes lower.

**Figure 6:**
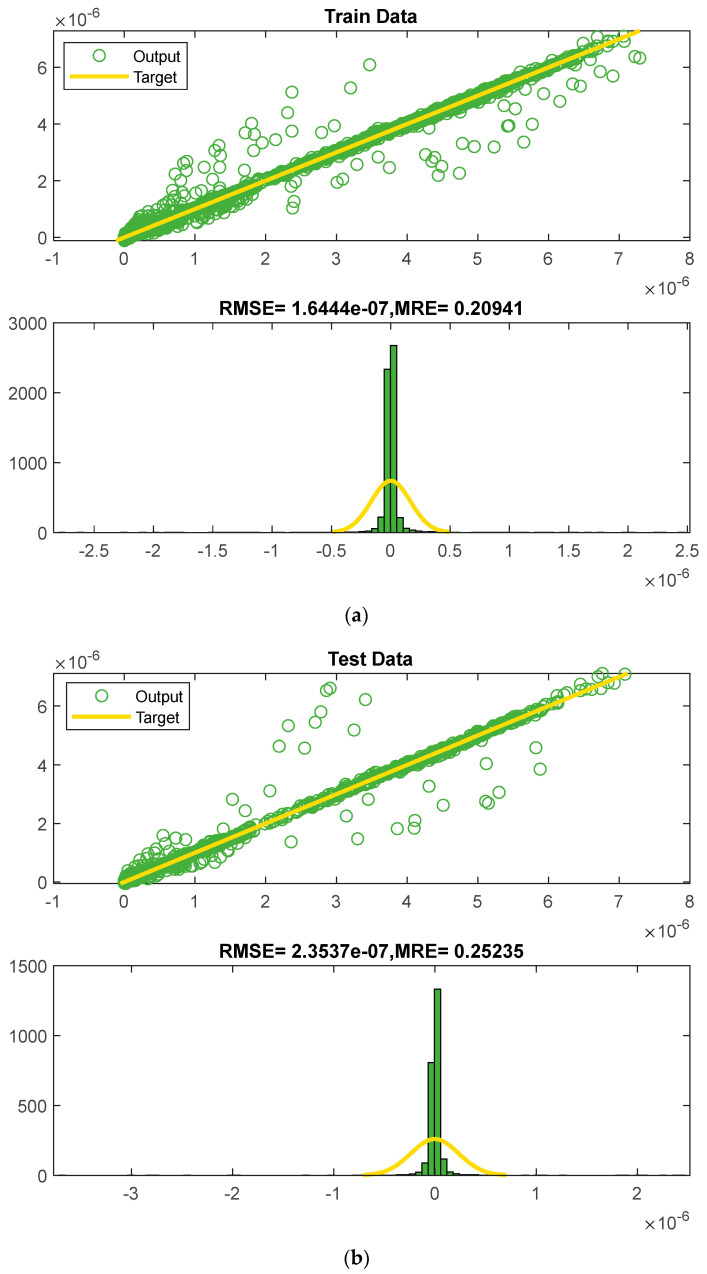
Diagram of the regression and error histograms for the: (**a**) training and (**b**) test datasets.

Figure 6 exhibits two error histograms and regression plots on the training and testing data to visually emphasize the neural network’s performance. In the regression graph, the green circle represents the neural network’s prediction and the yellow line represents the optimal answer (the value of air kerma generated using the MCNP algorithm). These coincide, proving the network’s high precision. Some input and output data of the GMDH neural network are displayed in Table 1. In this table, some of the tube voltage values and the location that is considered inputs of the network can be seen, along with the amount of air kerma calculated by the MCNP code. In this table, you can see the performance of the neural network in finding the input–output relationship. It should be noted that for the best possible performance of the neural network, first the inputs and outputs are normalized, and then after predicting the output, the data are returned to their initial state. The amount of air kerma predicted by the neural network is also provided. As it is clear, the value predicted by the neural network has a slight difference from the calculated value, which indicates the acceptable performance of the neural network.

## 5. Conclusions

Calculating air kerma using the MCNP code is very time-consuming and applies a high volume of calculations to the system, which requires relatively powerful processors to simulate the radiation field and calculate air kerma. For this purpose, in this research, an attempt has been made to provide a quick and low calculation method for predicting this parameter by calculating air kerma at limited points. The MCNP algorithm and a neural network were used in this research to find the air kerma in the radiation field of the X-ray tube. This was accomplished by inspecting the X-ray tube at a voltage range of 40 to 140 kV. Data from 1375 points throughout the radiation field of the X-ray tube were analyzed to determine the air kerma at each voltage. The generated data matrix contains the 8250 columns (various samples) and four rows (three location attributes and X-ray voltage) needed to construct the neural network. Given the location and voltage of the X-ray tube, the supervised, fast-learning GMDH network was trained to forecast air kerma. The suggested model exhibited an MRE of less than 0.25% for predicting air kerma. Due to its excellent precision and speed, it is the most accurate approach for determining the air kerma in the radiation field of an X-ray tube. While the approach employed in this work was specifically focused on estimating air kerma for a particular X-ray tube design (fixed target angle of 20°), it may be utilized for a broad range of X-ray tube radiation fields. The suggested approach may also be used to calculate other radiation characteristics, such as absorbed dose.

## Figures and Tables

**Table 1:** Some input and output data of the GMDH neural network.

ϕ (deg)	θ (deg)	R (mm)	V (kV)	Simulated Air Kerma (1 × e^−5^)	Predicted Air Kerma (1 × e^−5^)
0	0	250	40	0.3730	0.3699
2	0	250	40	0.3190	0.3272
4	0	250	40	0.3040	0.2977
6	0	250	40	0.2850	0.2757
8	0	250	40	0.2630	0.2593
10	0	250	40	0.2370	0.2414
12	0	250	40	0.2060	0.2111
14	0	250	40	0.1680	0.1650
16	0	250	40	0.1220	0.1148
18	0	250	40	0.0648	0.0718
0	15	500	40	0.0932	0.0934
2	15	500	40	0.0799	0.0788
4	15	500	40	0.0761	0.0749
6	15	500	40	0.0717	0.0698
8	15	500	40	0.0667	0.0640
10	15	500	40	0.0605	0.0598
12	15	500	40	0.0530	0.0540
14	15	500	40	0.0443	0.0426
16	15	500	40	0.0335	0.0289
18	15	500	40	0.0204	0.0194
20	15	500	40	0.0019	0.0031
0	30	750	40	0.0414	0.0408
2	30	750	40	0.0350	0.0361
4	30	750	40	0.0335	0.0370
6	30	750	40	0.0318	0.0344
8	30	750	40	0.0298	0.0289
10	30	750	40	0.0276	0.0253
12	30	750	40	0.0248	0.0249
14	30	750	40	0.0216	0.0244
16	30	750	40	0.0179	0.0214
18	30	750	40	0.0135	0.0165
20	30	750	40	0.0085	0.0066
0	45	1000	40	0.0233	0.0204
2	45	1000	40	0.0198	0.0181
4	45	1000	40	0.0191	0.0189
6	45	1000	40	0.0184	0.0198
8	45	1000	40	0.0175	0.0189
10	45	1000	40	0.0166	0.0171
12	45	1000	40	0.0154	0.0160
14	45	1000	40	0.0142	0.0160
16	45	1000	40	0.0128	0.0161
18	45	1000	40	0.0111	0.0150
20	45	1000	40	0.0026	0.0104
0	60	1250	40	0.0149	0.0135
2	60	1250	40	0.0139	0.0116
4	60	1250	40	0.0136	0.0111
6	60	1250	40	0.0132	0.0122
8	60	1250	40	0.0128	0.0129
10	60	1250	40	0.0124	0.0121
12	60	1250	40	0.0119	0.0105
14	60	1250	40	0.0114	0.0096
16	60	1250	40	0.0109	0.0096
18	60	1250	40	0.0102	0.0084
20	60	1250	40	0.0095	0.0028
0	0	250	60	0.3990	0.3975
2	0	250	60	0.3780	0.3801
4	0	250	60	0.3530	0.3544
6	0	250	60	0.3240	0.3247
8	0	250	60	0.2930	0.2961
10	0	250	60	0.2580	0.2651
12	0	250	60	0.2180	0.2218
14	0	250	60	0.1730	0.1636
16	0	250	60	0.1220	0.1034
18	0	250	60	0.0654	0.0562
0	15	500	60	0.0998	0.0974
2	15	500	60	0.0948	0.0920
4	15	500	60	0.0885	0.0889
6	15	500	60	0.0818	0.0821
8	15	500	60	0.0743	0.0742
10	15	500	60	0.0659	0.0673
12	15	500	60	0.0564	0.0577
14	15	500	60	0.0459	0.0415
16	15	500	60	0.0339	0.0239
18	15	500	60	0.0205	0.0141
20	15	500	60	0.0042	0.0030
0	30	750	60	0.0444	0.0457
2	30	750	60	0.0427	0.0408
4	30	750	60	0.0402	0.0399
6	30	750	60	0.0375	0.0375
8	30	750	60	0.0345	0.0338
10	30	750	60	0.0311	0.0312
12	30	750	60	0.0274	0.0293
14	30	750	60	0.0233	0.0250
16	30	750	60	0.0188	0.0182
18	30	750	60	0.0138	0.0122
20	30	750	60	0.0084	0.0047
0	45	1000	60	0.0250	0.0285
2	45	1000	60	0.0243	0.0247
4	45	1000	60	0.0231	0.0215
6	45	1000	60	0.0219	0.0205
8	45	1000	60	0.0206	0.0206
10	45	1000	60	0.0191	0.0208
12	45	1000	60	0.0176	0.0201
14	45	1000	60	0.0158	0.0182
16	45	1000	60	0.0139	0.0152
18	45	1000	60	0.0119	0.0121
20	45	1000	60	0.0040	0.0079
0	60	1250	60	0.0160	0.0154
2	60	1250	60	0.0158	0.0177
4	60	1250	60	0.0153	0.0163
6	60	1250	60	0.0147	0.0149
8	60	1250	60	0.0142	0.0143
10	60	1250	60	0.0135	0.0136
12	60	1250	60	0.0129	0.0124
14	60	1250	60	0.0121	0.0112
16	60	1250	60	0.0114	0.0106
18	60	1250	60	0.0105	0.0100
20	60	1250	60	0.0096	0.0077
0	0	250	80	0.4130	0.4087
2	0	250	80	0.4070	0.4051
4	0	250	80	0.3750	0.3739
6	0	250	80	0.3410	0.3326
8	0	250	80	0.3040	0.2938
10	0	250	80	0.2630	0.2587
12	0	250	80	0.2190	0.2180
14	0	250	80	0.1710	0.1641
16	0	250	80	0.1200	0.1029
18	0	250	80	0.0645	0.0490
0	15	500	80	0.1030	0.1027
2	15	500	80	0.1020	0.1040
4	15	500	80	0.0942	0.0983
6	15	500	80	0.0861	0.0862
8	15	500	80	0.0772	0.0732
10	15	500	80	0.0675	0.0633
12	15	500	80	0.0570	0.0546
14	15	500	80	0.0456	0.0423
16	15	500	80	0.0333	0.0278
18	15	500	80	0.0202	0.0183
20	15	500	80	0.0061	0.0082
0	30	750	80	0.0459	0.0453
2	30	750	80	0.0462	0.0443
4	30	750	80	0.0431	0.0445
6	30	750	80	0.0398	0.0423
8	30	750	80	0.0362	0.0372
10	30	750	80	0.0323	0.0315
12	30	750	80	0.0281	0.0266
14	30	750	80	0.0236	0.0217
16	30	750	80	0.0187	0.0170
18	30	750	80	0.0136	0.0144
20	30	750	80	0.0082	0.0094
0	45	1000	80	0.0258	0.0274
2	45	1000	80	0.0263	0.0254
4	45	1000	80	0.0249	0.0232
6	45	1000	80	0.0234	0.0229
8	45	1000	80	0.0218	0.0231
10	45	1000	80	0.0201	0.0219
12	45	1000	80	0.0183	0.0191
14	45	1000	80	0.0162	0.0155
16	45	1000	80	0.0141	0.0129
18	45	1000	80	0.0119	0.0119
20	45	1000	80	0.0054	0.0090
0	60	1250	80	0.0165	0.0154
2	60	1250	80	0.0167	0.0183
4	60	1250	80	0.0161	0.0173
6	60	1250	80	0.0154	0.0160
8	60	1250	80	0.0147	0.0151
10	60	1250	80	0.0140	0.0141
12	60	1250	80	0.0132	0.0124
14	60	1250	80	0.0123	0.0109
16	60	1250	80	0.0114	0.0106
18	60	1250	80	0.0105	0.0109
20	60	1250	80	0.0094	0.0094
0	0	250	100	0.4320	0.4285
2	0	250	100	0.4260	0.4248
4	0	250	100	0.3910	0.3876
6	0	250	100	0.3530	0.3424
8	0	250	100	0.3120	0.3028
10	0	250	100	0.2680	0.2694
12	0	250	100	0.2220	0.2328
14	0	250	100	0.1720	0.1816
16	0	250	100	0.1200	0.1156
18	0	250	100	0.0657	0.0503
0	15	500	100	0.1080	0.1099
2	15	500	100	0.1070	0.1105
4	15	500	100	0.0982	0.1016
6	15	500	100	0.0891	0.0888
8	15	500	100	0.0793	0.0762
10	15	500	100	0.0688	0.0668
12	15	500	100	0.0577	0.0593
14	15	500	100	0.0459	0.0487
16	15	500	100	0.0334	0.0332
18	15	500	100	0.0203	0.0186
20	15	500	100	0.0075	0.0073
0	30	750	100	0.0480	0.0437
2	30	750	100	0.0482	0.0445
4	30	750	100	0.0447	0.0450
6	30	750	100	0.0410	0.0437
8	30	750	100	0.0371	0.0388
10	30	750	100	0.0328	0.0320
12	30	750	100	0.0284	0.0258
14	30	750	100	0.0237	0.0209
16	30	750	100	0.0187	0.0173
18	30	750	100	0.0135	0.0153
20	30	750	100	0.0081	0.0100
0	45	1000	100	0.0270	0.0264
2	45	1000	100	0.0275	0.0262
4	45	1000	100	0.0259	0.0247
6	45	1000	100	0.0242	0.0243
8	45	1000	100	0.0224	0.0237
10	45	1000	100	0.0205	0.0214
12	45	1000	100	0.0185	0.0176
14	45	1000	100	0.0164	0.0140
16	45	1000	100	0.0142	0.0126
18	45	1000	100	0.0119	0.0129
20	45	1000	100	0.0069	0.0094
0	60	1250	100	0.0173	0.0179
2	60	1250	100	0.0175	0.0195
4	60	1250	100	0.0168	0.0192
6	60	1250	100	0.0160	0.0181
8	60	1250	100	0.0152	0.0166
10	60	1250	100	0.0144	0.0147
12	60	1250	100	0.0135	0.0127
14	60	1250	100	0.0125	0.0115
16	60	1250	100	0.0115	0.0116
18	60	1250	100	0.0105	0.0119
20	60	1250	100	0.0095	0.0083
0	0	250	120	0.4490	0.4500
2	0	250	120	0.4350	0.4375
4	0	250	120	0.3970	0.3968
6	0	250	120	0.3580	0.3535
8	0	250	120	0.3150	0.3137
10	0	250	120	0.2700	0.2756
12	0	250	120	0.2230	0.2338
14	0	250	120	0.1730	0.1809
16	0	250	120	0.1220	0.1145
18	0	250	120	0.0674	0.0488
0	15	500	120	0.1120	0.1165
2	15	500	120	0.1090	0.1112
4	15	500	120	0.1000	0.1001
6	15	500	120	0.0904	0.0903
8	15	500	120	0.0802	0.0805
10	15	500	120	0.0694	0.0700
12	15	500	120	0.0580	0.0589
14	15	500	120	0.0462	0.0453
16	15	500	120	0.0337	0.0277
18	15	500	120	0.0207	0.0108
20	15	500	120	0.0088	0.0034
0	30	750	120	0.0499	0.0468
2	30	750	120	0.0487	0.0462
4	30	750	120	0.0451	0.0447
6	30	750	120	0.0412	0.0440
8	30	750	120	0.0371	0.0414
10	30	750	120	0.0328	0.0360
12	30	750	120	0.0283	0.0295
14	30	750	120	0.0236	0.0231
16	30	750	120	0.0186	0.0175
18	30	750	120	0.0135	0.0133
20	30	750	120	0.0082	0.0080
0	45	1000	120	0.0280	0.0285
2	45	1000	120	0.0278	0.0285
4	45	1000	120	0.0261	0.0254
6	45	1000	120	0.0243	0.0234
8	45	1000	120	0.0224	0.0228
10	45	1000	120	0.0205	0.0222
12	45	1000	120	0.0185	0.0204
14	45	1000	120	0.0163	0.0174
16	45	1000	120	0.0141	0.0146
18	45	1000	120	0.0118	0.0126
20	45	1000	120	0.0085	0.0071
0	60	1250	120	0.0179	0.0190
2	60	1250	120	0.0180	0.0180
4	60	1250	120	0.0172	0.0178
6	60	1250	120	0.0163	0.0168
8	60	1250	120	0.0155	0.0153
10	60	1250	120	0.0146	0.0139
12	60	1250	120	0.0136	0.0130
14	60	1250	120	0.0127	0.0125
16	60	1250	120	0.0117	0.0122
18	60	1250	120	0.0106	0.0110
20	60	1250	120	0.0095	0.0057
0	0	250	140	0.4610	0.4537
2	0	250	140	0.4390	0.4354
4	0	250	140	0.4000	0.3975
6	0	250	140	0.3590	0.3586
8	0	250	140	0.3160	0.3161
10	0	250	140	0.2710	0.2689
12	0	250	140	0.2240	0.2218
14	0	250	140	0.1750	0.1763
16	0	250	140	0.1240	0.1262
18	0	250	140	0.0697	0.0725
0	15	500	140	0.1150	0.1178
2	15	500	140	0.1100	0.1099
4	15	500	140	0.1010	0.0996
6	15	500	140	0.0907	0.0931
8	15	500	140	0.0805	0.0840
10	15	500	140	0.0697	0.0697
12	15	500	140	0.0583	0.0548
14	15	500	140	0.0467	0.0430
16	15	500	140	0.0343	0.0321
18	15	500	140	0.0213	0.0201
20	15	500	140	0.0099	0.0121
0	30	750	140	0.0513	0.0496
2	30	750	140	0.0486	0.0492
4	30	750	140	0.0449	0.0455
6	30	750	140	0.0410	0.0439
8	30	750	140	0.0368	0.0420
10	30	750	140	0.0325	0.0376
12	30	750	140	0.0280	0.0312
14	30	750	140	0.0234	0.0249
16	30	750	140	0.0186	0.0197
18	30	750	140	0.0137	0.0165
20	30	750	140	0.0085	0.0130
0	45	1000	140	0.0288	0.0273
2	45	1000	140	0.0277	0.0282
4	45	1000	140	0.0260	0.0231
6	45	1000	140	0.0242	0.0191
8	45	1000	140	0.0222	0.0188
10	45	1000	140	0.0203	0.0208
12	45	1000	140	0.0183	0.0214
14	45	1000	140	0.0162	0.0184
16	45	1000	140	0.0140	0.0133
18	45	1000	140	0.0118	0.0098
20	45	1000	140	0.0100	0.0072
0	60	1250	140	0.0185	0.0190
2	60	1250	140	0.0183	0.0162
4	60	1250	140	0.0174	0.0165
6	60	1250	140	0.0166	0.0161
8	60	1250	140	0.0157	0.0151
10	60	1250	140	0.0147	0.0148
12	60	1250	140	0.0138	0.0147
14	60	1250	140	0.0128	0.0137
16	60	1250	140	0.0118	0.0120
18	60	1250	140	0.0107	0.0111
20	60	1250	140	0.0097	0.0109

## Data Availability

The data presented in this study are available on request from the corresponding author.
